# Draft Genome Sequence of Mangrove-Derived *Streptomyces* sp. MUSC 125 with Antioxidant Potential

**DOI:** 10.3389/fmicb.2016.01470

**Published:** 2016-09-15

**Authors:** Hooi-Leng Ser, Wen-Si Tan, Nurul-Syakima Ab Mutalib, Wai-Fong Yin, Kok-Gan Chan, Bey-Hing Goh, Learn-Han Lee

**Affiliations:** ^1^Novel Bacteria and Drug Discovery Research Group, School of Pharmacy, Monash University MalaysiaBandar Sunway, Malaysia; ^2^Division of Genetics and Molecular Biology, Faculty of Science, Institute of Biological Sciences, University of MalayaKuala Lumpur, Malaysia; ^3^UKM Medical Molecular Biology Institute, UKM Medical Centre, Universiti Kebangsaan MalaysiaKuala Lumpur, Malaysia; ^4^Center of Health Outcomes Research and Therapeutic Safety (Cohorts), School of Pharmaceutical Sciences, University of PhayaoPhayao, Thailand

**Keywords:** genome sequence, mangrove, *Streptomyces*, antioxidant, bioinformatics

## Introduction

Microorganisms remain as one of the most important source of pharmaceutically important drugs (Berdy, 2005; Debbab et al., 2010; Waditee-Sirisattha et al., 2016). Among the bacteria domain, *Streptomyces* genus has received considerable attention by the Scientific community for its seemingly unmatched capability of producing useful bioactive metabolites. The *Streptomyces* genus was initially proposed by Waksman and Henrici (1943); as the largest genus of *Actinobacteria*, it is comprised of over 780 species with validly published names (http://www.bacterio.cict.fr/). These gram-positive bacteria have contributed remarkably in natural product discovery as they synthesize compounds with diverse chemical structures and biological activities such as anticancer, antibacterial, antioxidant, antifungal, and immunosuppresants activities (Berdy, 2005; Gallagher et al., 2010; Manivasagan et al., 2013; Ser et al., 2015a, 2016a).

Over the years, drug screening programs have been focusing on identification of terrestrial microorganisms and investigation of their bioactive potential (Burg et al., 1979; Marcus et al., 1999). However, these efforts have resulted in rediscovery of the known bioactive compounds. Thus, researchers begun to venture into new and/or underexplored areas in the hope of discovering novel, potent bioactive metabolites. The mangrove ecosystem represents one of the world's most dynamic environments that produces commercial forest products, supports coastal fisheries and protects coastlines (Alongi, 2008). Factors such as fluctuations in salinity and tidal gradient are believed to be the driving force for metabolic adaptations, which could in turn lead to production of valuable metabolites. In fact, the growing interest in bioactive potentials of mangrove-derived *Streptomyces* has been demonstrated by the isolation of *Streptomyces pluripotens* (Lee et al., 2014a), *S. fradiae* (Prakash et al., 2015), *S. cheonanensis* (Mangamuri et al., 2016), and *S. malaysiense* (Ser et al., 2016b).

For this study, *Streptomyces* sp. MUSC 125 was initially isolated from mangrove soil in the east coast of Peninsular Malaysia (Lee et al., 2014b). As an attempt to explore the antioxidant capacity of MUSC 125, metal-chelating assay was performed and discovered its potential in quenching ferrous ions with activity ranging from 10.02 to 51.14% (unpublished data). Thus, the strain was selected for whole genome sequencing to obtain further understanding on its genomic potential.

## Materials and methods

### Isolation and culture of strain MUSC 125

*Streptomyces* sp. MUSC 125 was isolated from Tanjung Lumpur mangrove forest located in the city of Kuantan, State of Pahang, in December of the year 2012 (Lee et al., 2014b). Purified cultures were maintained on ISP medium 2 slants at room temperature for short-term storage and as glycerol suspensions (20%, v/v) at −80°C for long-term storage.

### Genome sequencing and bioinformatics analysis of MUSC 125

The genomic DNA of MUSC 125 was extracted with Masterpure™ DNA purification kit (Epicentre, Illumina Inc., Madison, WI, USA) followed by RNase (Qiagen, USA) treatment (Ser et al., 2015b). Quality of the extracted DNA was examined using NanoDrop spectrophotometer (Thermo Scientific, Waltham, MA, USA) and a Qubit version 2.0 fluorometer (Life Technologies, Carlsbad, CA, USA). Subsequently, DNA library was prepared using Nextera™ DNA Sample Preparation kit (Nextera, USA), while the library quality was validated by Bioanalyzer 2100 high sensitivity DNA kit (Agilent Technologies, Palo Alto, CA) prior to sequencing. The genome of strain MUSC 125 was sequenced on MiSeq platform with MiSeq Reagent Kit 2 (2 × 250 bp; Illumina Inc., Madison, WI, USA). Following that, the paired-end reads were trimmed and *de novo* assembled with CLC Genomics Workbench version 5.1 (CLC bio, Denmark). Gene prediction was performed using Prodigal version 2.6, whereas rRNA and tRNA were predicted using RNAmmer and tRNAscan SE version 1.21 (Lowe and Eddy, 1997; Lagesen et al., 2007; Hyatt et al., 2010). The assembly was uploaded for annotation to Rapid Annotation using Subsystem Technology (RAST) (Aziz et al., 2008).

## Results

**Figure 1:**
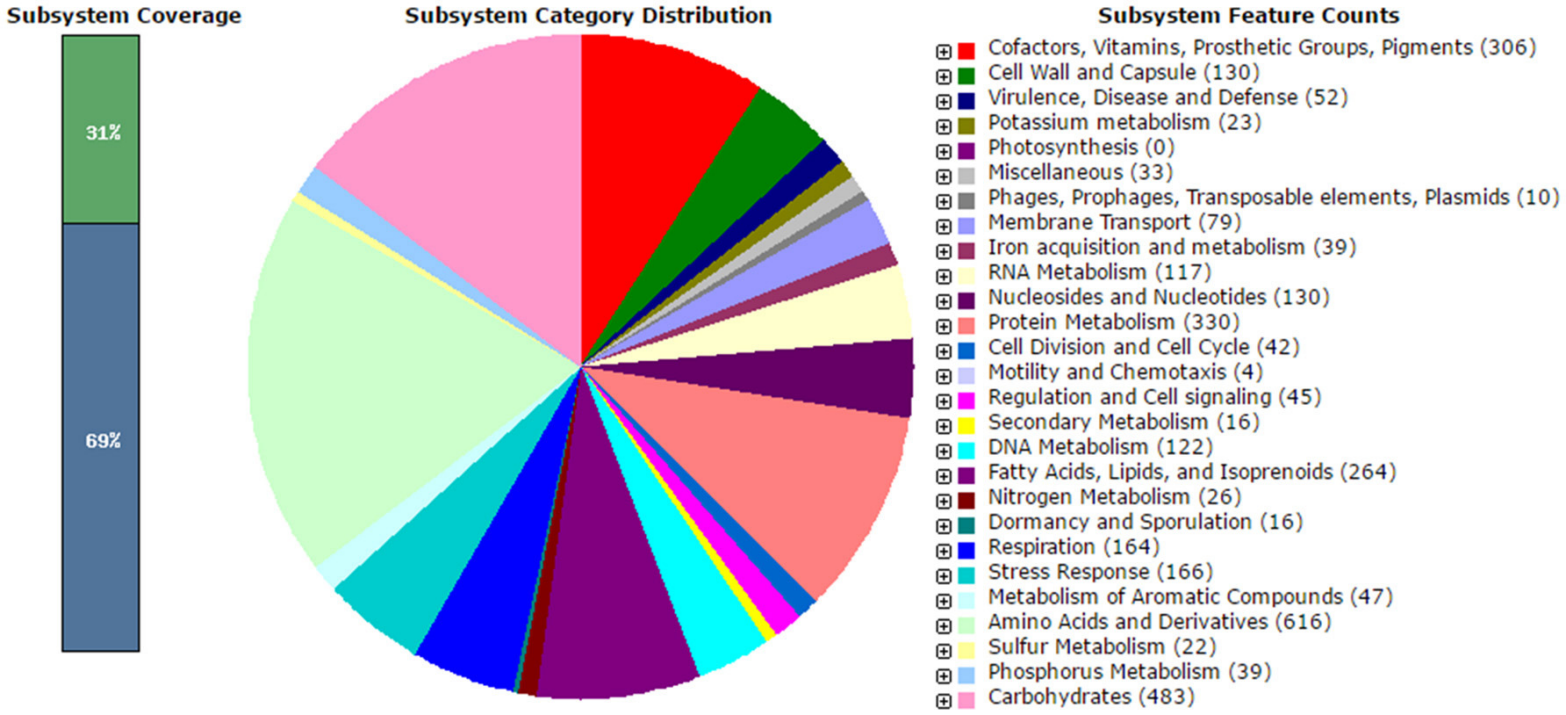
**Annotation of MUSC 125 genome using Rapid Annotation using Subsystem Technology (RAST)**.

Sequencing of MUSC 125 genome using Illumina technology generated a total of 4,518,422 reads. After adapter trimming, the reads were *de novo* assembled into 164 contigs using CLC Genomics workbench. The genome size of MUSC 125 is 7,656,461 bp with G + C content of 70.00% (Table 1). The whole genome project of MUSC 125 was deposited at DDBJ/EMBL/GenBank under accession number JUIG00000000 and the version described in this paper is the first version (JUIG01000000). The analyses of the draft genome identified 5991 open reading frames (ORFs), 68 tRNAs, and 4 rRNA (5S, 16S, 23S rRNA). The RAST annotation has assigned these genes into 419 subsystems, with maximum number of genes associated with amino acids and derivatives metabolism (8.84%), followed by carbohydrates (6.93 %) and protein metabolism subsystems (4.73%) (Figure 1).

**Table 1:** **General features of ***Streptomyces*** sp. MUSC 125 draft genome**.

	***Streptomyces* sp. MUSC 125**
Genome size (bp)	7,656,461
G+C content (%)	70.00
Genome coverage	94.0x
Contigs	164
Contigs N_50_ (bp)	139,282
Number of ORFs	5991
tRNA genes	68
rRNA genes (5S, 16S, 23S)	4
Bioproject ID	PRJNA261099
Biosample ID	SAMN03070123
Genome accession	JUIG00000000

### Direct link to deposited data and information to users

The genome sequence of *Streptomyces* sp. MUSC 125 (Biosample ID: SAMN03070123) can be accessed at NCBI using the accession number JUIG00000000. The genome project data are also available at GenBank under the genome Bioproject ID PRJNA261099. Users can download and use the data freely for research purpose only with acknowledgement to us and citing this paper as reference to the data.

## Author contributions

The experiments, data analysis and manuscript writing were performed by H-LS and W-ST, while N-SA, W-FY, K-GC, B-HG, and L-HL. provided vital guidance and technical support. L-HL founded the research project.

### Conflict of interest statement

The authors declare that the research was conducted in the absence of any commercial or financial relationships that could be construed as a potential conflict of interest.

## References

[B1] Alongi D. M. (2008). Mangrove forests: resilience, protection from tsunamis, and responses to global climate change. Estuar. Coast. Shelf Sci. 76, 1–13. 10.1016/j.ecss.2007.08.024

[B2] Aziz R. K. Bartels D. Best A. A. DeJongh M. Disz T. Edwards R. A.. (2008). The RAST Server: rapid annotations using subsystems technology. BMC Genomics 9:75. 10.1186/1471-2164-9-75 18261238 PMC2265698

[B3] Berdy J. (2005). Bioactive microbial metabolites. J. Antibiot. 58, 1–26. 10.1038/ja.2005.1 15813176

[B4] Burg R. W. Miller B. M. Baker E. E. Birnbaum J. Currie S. A. Hartman R.. (1979). Avermectins, new family of potent anthelmintic agents: producing organism and fermentation. Antimicrob. Agents Chemother. 15, 361–367. 10.1128/AAC.15.3.361 464561 PMC352666

[B5] Debbab A. Aly A. H. Lin W. H. Proksch P. (2010). Bioactive compounds from marine bacteria and fungi. Microb. Biotech. 3, 544–563. 10.1111/j.1751-7915.2010.00179.x 21255352 PMC3815768

[B6] Gallagher K. A. Fenical W. Jensen P. R. (2010). Hybrid isoprenoid secondary metabolite production in terrestrial and marine actinomycetes. Curr. Opin. Biotechnol. 21, 794–800. 10.1016/j.copbio.2010.09.010 20951024

[B7] Hyatt D. Chen G. L. Locascio P. F. Land M. L. Larimer F. W. Hauser L. J. (2010). Prodigal: prokaryotic gene recognition and translation initiation site identification. BMC Bioinformatics 11:119. 10.1186/1471-2105-11-119 20211023 PMC2848648

[B8] Lagesen K. Hallin P. Rodland E. A. Staerfeldt H. H. Rognes T. Ussery D. W. (2007). RNAmmer: consistent and rapid annotation of ribosomal RNA genes. Nucleic Acids Res. 35, 3100–3108. 10.1093/nar/gkm160 17452365 PMC1888812

[B9] Lee L. H. Zainal N. Azman A. S. Eng S. K. Ab Mutalib N. S. Yin W. F.. (2014a). *Streptomyces pluripotens* sp. nov., a bacteriocin-producing streptomycete that inhibits meticillin-resistant *Staphylococcus aureus*. Int. J. Syst. Evol. Microbiol. 64, 3297–3306. 10.1099/ijs.0.065045-0 24994773

[B10] Lee L. H. Zainal N. Azman A. S. Eng S. K. Goh B. H. Yin W. F. (2014b). Diversity and antimicrobial activities of actinobacteria isolated from tropical mangrove sediments in Malaysia. Sci. World J. 698178, 1–14. 10.1155/2014/698178 PMC4138949 25162061

[B11] Lowe T. M. Eddy S. R. (1997). tRNAscan-SE: a program for improved detection of transfer RNA genes in genomic sequence. Nucleic Acids Res. 25, 955–964. 9023104 10.1093/nar/25.5.955 PMC146525

[B12] Mangamuri U. Muvva V. Poda S. Naragani K. Munaganti R. K. Chitturi B.. (2016). Bioactive metabolites produced by *Streptomyces cheonanensis* VUK-A from Coringa mangrove sediments: isolation, structure elucidation and bioactivity. 3 Biotech 6, 1–8. 10.1007/s13205-016-0398-6 28330133 PMC4752944

[B13] Manivasagan P. Venkatesan J. Sivakumar K. Kim S. K. (2013). Production, characterization and antioxidant potential of protease from *Streptomyces* sp. MAB18 using poultry wastes. BioMed Res. Int. 2013:496586. 10.1155/2013/496586 23991418 PMC3749541

[B14] Marcus N. H. Pomponi S. Rhines P. Tester P. Vena J. (1999). Marine derived pharmaceuticals and related bioactive compounds, in Monsoons to Microbes: Understanding the Oceanss Role in Human Health, eds Fiedler H. P. Brunter C. Riedlinger J. Bull A. T. Knutsen G. Goodfellow (Washington, DC: National Academies Press), 7186.

[B15] Prakash S. Ramasubburayan R. Iyapparaj P. Arthi A. P. R. Ahila N. K. Ramkumar V. S. (2015). Environmentally benign antifouling potentials of triterpene-glycosides *from Streptomyces fradiae*: a mangrove isolate. RSC Adv. 5, 29524–29534. 10.1039/C4RA15335A

[B16] Ser H. L. Palanisamy U. D. Yin W. F. Abd Malek S. N. Chan K. G. Goh B. H.. (2015a). Presence of antioxidative agent, Pyrrolo[1,2-a]pyrazine-1,4- dione, hexahydro- in newly isolated *Streptomyces mangrovisoli* sp. nov. Front. Microbiol. 6:854. 10.3389/fmicb.2015.00854 26347733 PMC4542459

[B17] Ser H. L. Palanisamy U. D. Yin W. F. Chan K. G. Goh B. H. Lee L. H. (2016b). *Streptomyces malaysiense* sp. nov., a novel Malaysian mangrove soil actinobacterium with antioxidative activity and cytotoxic potential against human cancer cell lines. Sci. Rep. 6:24247. 10.1038/srep24247 27072394 PMC4829849

[B18] Ser H. L. Tan L. T. H. Palanisamy U. D. Abd Malek S. N. Yin W. F. Chan K. G.. (2016a). *Streptomyces antioxidans* sp. nov., a novel mangrove soil actinobacterium with antioxidative and neuroprotective potentials. Front. Microbiol. 7:899. 10.3389/fmicb.2016.00899 27379040 PMC4909769

[B19] Ser H. L. Tan W. S. Ab Mutalib N. S. Cheng H. J. Yin W. F. Chan K. G.. (2015b). Genome sequence of *Streptomyces pluripotens* MUSC 135^T^ exhibiting antibacterial and antioxidant activity. Mar. Gen. 24, 281–283. 10.1016/j.margen.2015.09.010 26452302

[B20] Waditee-Sirisattha R. Kageyama H. Takabe T. (2016). Halophilic microorganism resources and their applications in industrial and environmental biotechnology. AIMS Microbiol. 2, 42–54. 10.3934/microbiol.2016.1.42

[B21] Waksman S. A. Henrici A. T. (1943). The nomenclature and classification of the actinomycetes. J. Bacteriol. 46, 337–341. 16560709 10.1128/jb.46.4.337-341.1943 PMC373826

